# Transient cognitive impacts of oxygen deprivation caused by catch-and-release angling

**DOI:** 10.1098/rsbl.2024.0527

**Published:** 2025-01-15

**Authors:** Henrik Flink, Adrian Berge, Francesca Leggieri, Niclas Kolm, Petter Tibblin

**Affiliations:** ^1^ Centre for Ecology and Evolution in Microbial Model Systems (EEMiS), Department of Biology and Environmental Science, Faculty of Health and Life Sciences, Linnaeus University, Kalmar 39231, Sweden; ^2^ River Ecology and Management Research Group RivEM, Department of Environmental and Life Sciences, Karlstad University, Karlstad, Sweden; ^3^ Department of Zoology, Stockholm University, Svante Arrhenius väg 18 B, Stockholm 106 91, Sweden

**Keywords:** brain function, working memory, recreational fishing, hypoxia, Y-maze, rainbow trout

## Abstract

Vertebrate brain function is particularly sensitive to the effects of hypoxia, with even brief periods of oxygen deprivation causing significant brain damage and impaired cognitive abilities. This study is the first to investigate the cognitive consequences of hypoxia in fish, specifically induced by exhaustive exercise and air exposure, conditions commonly encountered during catch-and-release (C&R) practices in recreational fishing. Angling exerts substantial pressure on inland fish populations, underscoring the need for sustainable practices like C&R. While C&R survival rates are generally high, understanding its sublethal impacts is crucial for evaluating the practice’s ethical and ecological sustainability. We examined the effects of these stressors on the cognitive function of 238 rainbow trout, using the free movement pattern Y-maze method to assess working memory through navigational search patterns during free exploration sessions. Our results showed that air exposure led to short-term (3–4 h post-treatment), but transient impairments in working memory, with no long-term cognitive deficits observed at one week and one month post-treatment. These findings emphasize the high tolerance of fish to hypoxia and support the sustainability of C&R as a tool in fisheries management.

## 1. Introduction

Global overfishing, driven by both commercial and recreational practices, has resulted in widespread declines in fish populations, destabilized ecosystems and poses significant threats to the sustainability of aquatic resources [1–8]. To mitigate the overexploitation of fish stocks in recreational fisheries, catch-and-release (C&R) angling, where captured fish are returned to the water, has emerged as a widely adopted management strategy [9–12]. For C&R to serve as a truly sustainable and ethical alternative to catch-and-keep practices, ensuring the survival and well-being of released fish is crucial. However, C&R exposes fish to various stressors, including the exertion during landing, which often triggers a comprehensive stress response that challenges their physiological limits [9,13–15]. Exhaustive exercise increases tissue oxygen consumption and elevates the demand for oxygen, often leading to anaerobic metabolism, which necessitates recovery time with increased ventilation to restore normal function [13]. Yet, during this recovery period, many fish subjected to C&R are exposed to acute hypoxia when removed from the water for handling activities such as unhooking, measuring, admiration or photography [16]. This exposure leads to additional challenges, including lactate accumulation, increased acidosis, exacerbated osmoregulatory disruption and poses a risk for anoxia, which could potentially lead to cell death through necrosis or apoptosis, and in the worst case, result in the fish’s death [17–19]. While numerous studies indicate that mortality rates following C&R can be minimized with best practices, such as limiting air exposure [9,10,17], there remains significant concern regarding the sublethal risks associated with brief air exposure post-exhaustion, including impacts on growth, reproduction and behaviour [16,17,20,21].

Given the physiological challenges caused by C&R, a potentially important yet so far overlooked aspect is the impact of C&R on fish neurobiology, particularly concerning brain function and cognition. The vertebrate brain, with its high intrinsic rate of oxygen consumption, is typically among the first organs to fail in anoxia [22,23]. It is well established that mammals experience rapid brain damage and impaired cognitive function, including long-term effects, when deprived of oxygen [24]. While there is significant variation in brain function and morphology among fish species, many exhibit a relative brain metabolic rate comparable to that of mammals [25]. Studies on rainbow trout (*Oncorhynchus mykiss*) suggest that their sensitivity to anoxia matches that of mammals [26]. When deprived of oxygen, their brains rapidly lose ion homeostasis and release glutamate, processes also observed in mammalian acute neuronal injury arising from brain ischaemia [26,27]. However, fish possess a superior capacity to repair brain damage due to their more plastic brains, characterized by a higher turnover of cells and the ability to produce new axons and neurons [19,28]. Determining whether the exhaustive exercise and air exposure typical in C&R angling can induce negative neurological effects in fish is crucial, as this knowledge is essential for ensuring sustainable C&R practices.

Evaluating brain damage and cognitive impairment in fish subjected to C&R poses significant methodological challenges. In this study, we took advantage of the recently established free movement pattern (FMP) Y-maze method, which effectively assesses working memory impairment in fish without necessitating rule learning, extensive handling, repeated manipulation or reinforcement [29–32]. This approach thus marks a notable advancement over previous methods such as the two-choice task and continuous Y-maze task. In the FMP Y-maze, fish are allowed 1 h of free exploration, during which the sequence of left and right turns made by the fish is recorded and analysed. By extending the observation period to 1 h, the FMP Y-maze method focuses on navigational search patterns rather than responses driven purely by novelty [29]. Furthermore, the method includes a habituation phase that effectively reduces anxiety responses. The turns recorded are grouped into overlapping sequences of four, known as tetragrams, to identify repeated patterns in navigational behaviour. There are a total of 16 possible tetragram combinations. The inclusion of prior choices within these tetragrams enables analysis of navigational strategies and provides insight into the fish’s decision-making process [29]. As tetragrams capture information on previous turns, they reflect how prior choices influence subsequent behaviour. Among these, the specific sequences ‘left–right–left–right (LRLR)’ and ‘right–left–right–left (RLRL)’ represent alternation patterns, where a fish switches direction at each turn (i.e. LRLR or RLRL). Previous research indicates that zebrafish (*Danio rerio*), similar to humans and rodents, predominantly use these alternations as a search strategy [29–34]. This behaviour is considered a reliable proxy for working memory [29]. Notably, pharmaceuticals known to impair working memory have been shown to reduce the proportion of these alternations, reinforcing the method’s reliability in detecting cognitive impairments [29].

To explore the potential impacts of C&R on cognitive function, we conducted experiments on rainbow trout, a species with high oxygen demands and commonly targeted in recreational fisheries. The fish underwent simulated C&R procedures, which included combinations of exhaustive exercise and air exposure for 30 or 60 s, as well as treatments involving only exhaustive exercise or air exposure. A control group was maintained without exposure to any of these stressors. We evaluated working memory using the FMP Y-maze at three post-treatment intervals: 3−4 h (short-term), one week (intermediate) and one month (long-term). We hypothesized that any C&R-induced effects on working memory would be reflected by a reduced proportion of alternations, particularly in fish subjected to both exhaustion and extended air exposure, with the most pronounced effects expected in the short term owing to the potential for cognitive recovery over time.

## 2. Material and methods

### (a) Fish housing

We investigated how C&R stress affects cognitive function in hatchery-raised rainbow trout (*n* = 238, total length 18.1 ± 1.4 cm, mean ± s.d., Hultsby fish farm, Sweden). The study took place at Linnaeus University, Kalmar, Sweden, from 5 September to 27 November 2023. Fish were kept in five 750 l tanks with flowing brackish water (~7 psu) from the Baltic Sea, maintained at a constant 14°C. Light cycles were set at 12 h of light and 12 h of darkness, and fish were fed pellets (INICIO Plus 2.0 mm, BioMar A/S, Brande, Denmark) once daily, equivalent to 1% of their estimated mean body weight.

### (b) Fish tagging

Fish were tagged individually with passive integrated transponders (PIT tags, 12 mm long, 2.12 mm diameter, model HDX12, Biomark, Boise, Idaho, USA) using a handheld MK25 implant gun (Biomark) approximately five weeks before simulated C&R treatments and initial working memory assessment. Prior to tagging, fish were anaesthetized in 60 mg l^−1^ tricaine methanesulfonate (MS-222, Sigma-Aldrich, St Louis, Missouri, USA) buffered with sodium bicarbonate until fully immobilized, ensuring no reaction to handling. It is important to note that MS-222 sedation does not impact working memory long-term (after 3 days), as evaluated by the FMP Y-maze [32]. All fish handling, including tagging and transfer, was conducted with fish submerged to minimize stress and air exposure.

### (c) Simulated catch-and-release treatment

The C&R stress was simulated using well-established, standardized and controlled protocols, enabling a clear distinction between the individual effects of exhaustion, oxygen deprivation and their combined interactions [16,18,35]. To achieve this, fish were randomly allocated to one of five experimental treatments, ensuring stratified sampling based on body length. The treatments were as follows: control (*n* = 52), exhaustive exercise (*n* = 46), 60 s air exposure (*n* = 53), exhaustive exercise followed by 30 s air exposure (*n* = 42) and exhaustive exercise followed by 60 s air exposure (*n* = 45). Each fish was individually netted, scanned with a handheld PIT-tag reader to determine its pre-assigned treatment, and then exposed to its respective treatment before being released into a tank with mixed treatments. For the exhaustive exercise, each fish was placed alone in a treatment tank (L80 × W65 × D45 cm) and manually chased by hand until exhaustion, signalled by the fish being caught by the caudal fin five times (mean chasing time 82 ± 22 s). The air exposure treatment involved holding each fish out of water in a dip net for either 30 or 60 s. To evaluate the immediate stress response following different treatments, we observed the fish after release, recording any loss of balance and recovery times. No mortality occurred in the first week after treatments.

### (d) Assessment of working memory with the free movement pattern Y-maze

Working memory was assessed in eight identical Y-mazes over three 1 h exploration trials per fish at varying time intervals post-treatment: between 3 and 4 h (short-term, chosen to capture peak lactate levels and maximum physiological stress response [18]), one week (intermediate) and one month (long-term). We adapted the FMP Y-maze method for zebrafish [29] to fit the larger size of rainbow trout, adjusting the maze dimensions to L50 × W20 × H20 cm, where the length (L) represents the arm-length of the maze. These mazes maintained equal-arm proportions to remove intra-maze cues but retained extra-maze cues for orientation, including diffuse light sources. Trials were conducted under ambient light with a maximum level of 2 lx, and the presence of the experimenter was minimized to reduce stress and distraction. Water was exchanged after every two trials, and fish movements were recorded using IR cameras. Due to technical issues, video data for 24 short-term and eight intermediate trials were recorded during the second hour of a 2 h free exploration period in the Y-maze, shifting the testing time for some short-term trials to 4−5 h post-treatment, instead of the intended 3−4 h. These trials were randomly distributed across treatments. A separate analysis (results not shown), which considered whether the fish were recorded during the second hour instead of the first, confirmed that their inclusion did not affect the overall results.

**Figure 1.**
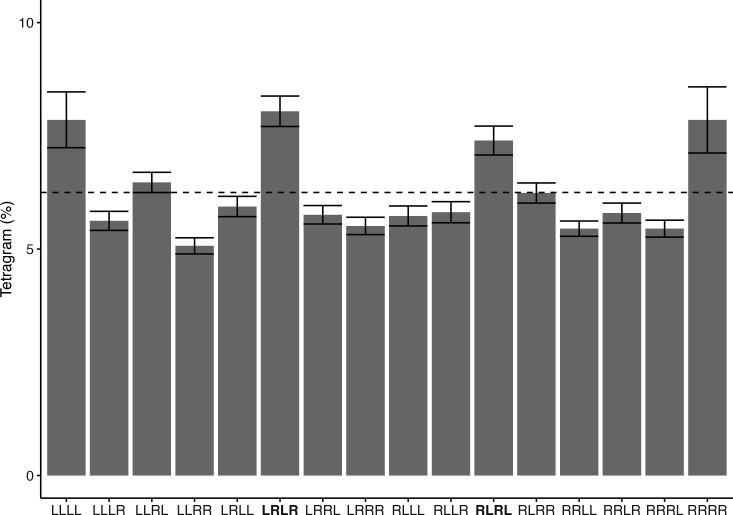
Frequency distribution of tetragram strategy (mean and s.e.m.) for 1 h exploration in the FMP Y-maze by fish from control treatment tested 3−4 h, one week and one month after treatment (*n* = 119). The common search strategy of alternations (LRLR and RLRL) is highlighted in bold. The dashed lines indicate a random strategy at 6.25%.

Fish movement in each video was tracked automatically using the trackPath function in the R package pathtrackr [36]. Pixel coordinates representing fish positions were recorded and linked to specific maze regions, then recoded to determine the occupied maze arm using a reference image with arm polygons. Each fish’s tracked path was plotted to verify tracking accuracy, and manual tracking was adopted when automatic tracking was insufficient. Transitions between arms were converted into sequences of left and right turns and organized into 16 overlapping tetragrams (figure 1, see electronic supplementary material, figures S1–S5 for frequency distributions of tetragram strategy grouped by treatment and time interval). Thirteen trials were excluded as the fish performed fewer than four turns and therefore could not be organized into tetragrams. Additionally, 40 fish succumbed prior to the intermediate trials due to an accidental laboratory incident, and 16 fish perished sporadically prior to the long-term trials for unknown reasons. Data from prior trials for these fish remain in the analysis.

### (e) Data analyses

**Figure 2.**
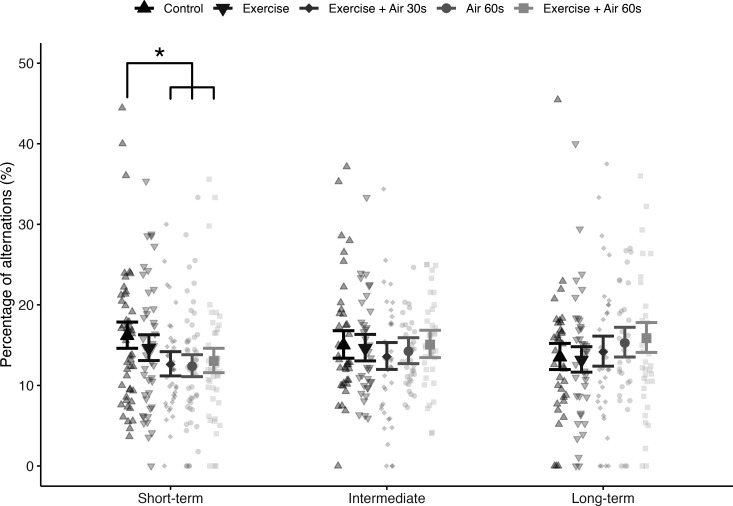
Percentage of alternations used in the tetragram strategy during a 1 h exploration in the FMP Y-maze, categorized by simulated C&R treatments and time intervals. The means and 95% confidence intervals (large points and error bars) are predicted from the GLMM. Small points represent individual raw data, with two values omitted since they exceeded the scale range. Pairwise comparisons of all treatment and time interval combinations revealed a significantly lower use of alternation in the three treatment groups that included air exposure compared with the control group in the short-term interval (highlighted by *). No other significant differences were observed.

To evaluate the impact of C&R on working memory, we analysed the proportional use of alternations during 1 h exploration periods for each treatment group and time interval. A generalized linear mixed model (GLMM) was fitted using the glmmTMB package [37] with a binomial distribution and logit-link function, employing maximum likelihood estimation through Template Model Builder (TMB). The response variable was the proportion of successful alternations (the combined tetragrams LRLR and RLRL) out of the total tetragrams completed for each fish, represented in the model as the number of alternations and the number of non-alternations for each individual. The fixed explanatory variables included treatment groups, time interval and their interaction. To account for the hierarchical structure of the data, a random effect of fish ID was included. Additionally, the total number of turns completed was standardized (centred and scaled) to avoid singular fits and was incorporated as a covariate to control for potential activity level differences among treatment groups. The syntax for the R model was: glmmTMB(cbind(alternations, non_alternations) ~treatment * time_interval + completed_turns + (1 | fish_ID), family = binomial(link = 'logit')). Model diagnostics using the DHARMa package [38] indicated that residuals were appropriate, with no significant deviations from uniformity or signs of overdispersion. Predictions adjusted for non-focal terms (covariate and random effects) were computed using the Effect() function in the effects package [39] and visualized in figure 2 with the ggplot2 package [40]. *Post hoc* pairwise comparisons of estimated marginal means were conducted using the emmeans package [41] with estimated coefficients, standard errors and *p*-values adjusted using the Tukey method for multiple comparisons.

## 3. Results

Immediate post-release behaviour varied by treatment, indicating differing stress impacts. Control fish maintained a stable vertical orientation, while fish from other treatments displayed stress through temporary loss of balance. Specifically, exhaustive exercise led to 7% of fish showing balance loss, with a mean recovery time of 2.3 ± 0.58 s; exhaustive exercise followed by 30 s air exposure resulted in 30% with 3.1 ± 1.4 s; 60 s air exposure had 68% with 11.4 ± 19.4 s; and exhaustive exercise followed by 60 s air exposure saw 91% affected with recovery times averaging 54.6 ± 63.1 s. Fish in the control treatment employed an exploration strategy that was overrepresented by non-random sequences of alternating left and right turns (LRLR and RLRL), as well as repeated turns (left–left–left–left (LLLL) and right–right–right–right (RRRR)) (figure 1). The GLMM revealed a significant interaction between the simulated C&R treatment and time interval (*p* < 0.05), indicating that the effect of treatment on working memory (i.e. alternation usage) depends on the time elapsed since treatment (figure 2). Detailed model outputs, including estimates and confidence intervals for the interaction terms, are provided in electronic supplementary material, table S1. To further investigate this significant interaction effect, *post hoc* pairwise comparisons of estimated marginal means were conducted for all possible combinations. These comparisons showed that, in the short-term interval, the three treatments involving air exposure resulted in a significantly lower proportion of alternations compared with the control group (exhaustive exercise followed by 30 s air exposure versus control: estimate = 0.29, s.e. = 0.09, *p* = 0.01; 60 s air exposure vs control: estimate = 0.31, s.e. = 0.09, *p* = 0.003, and exhaustive exercise followed by 60 s air exposure versus control: estimate = 0.25, s.e. = 0.09, *p* = 0.04). In contrast, no significant differences were observed between treatments during the intermediate and long-term intervals. A full list of pairwise comparisons and their associated *p*-values can be found in electronic supplementary material, table S2.

## 4. Discussion

Understanding the sublethal effects of C&R practices is crucial for sustainable recreational fishing that supports fish welfare, viable fish stocks and functional ecosystems. This study investigated the effects of exhaustive exercise and air exposure, simulating C&R, on working memory in rainbow trout in both the short- and long-term. Results from the FMP Y-maze indicated that cognitive function is generally resilient to C&R, with no impairment in the use of behavioural alternations, our proxy for working memory, observed either one week or one month after treatment (figure 2). This suggests that rainbow trout can recover from the immediate and acute stress of C&R without long-term cognitive deficits. However, trials conducted 3−4 h after treatment revealed that air exposure caused a significant decrease in the use of alternations, indicating a short-term impairment of executive function and delayed recovery (figure 2). These findings highlight that while long-term cognitive impacts are minimal, acute effects immediately following air exposure could influence behaviour and potentially affect survival and fitness if fish are released back into the wild during this vulnerable period.

There was no observable additive effect of exhaustion combined with air exposure on working memory in our study. This finding contrasts with previous research suggesting that the combination of multiple stressors could have a synergistic effect on stress physiology in fish. One possible explanation for this discrepancy is that exhaustion and air exposure elicit different physiological responses: exhaustion primarily induces a stress response related to physical exertion, whereas air exposure triggers an anoxic stress response due to lack of oxygen [42]. Rainbow trout, which are adapted to oxygen-rich, cold-water environments, are particularly sensitive to hypoxia [26]. This sensitivity to low oxygen conditions may explain why air exposure alone was sufficient to impair working memory, regardless of prior exhaustion. In our study, the impairment observed appears to be primarily driven by the hypoxic stress of air exposure, overshadowing any additional effects from physical exhaustion.

Numerous studies have documented non-lethal short-term effects of C&R on various fish behaviours, such as equilibrium maintenance [42–44], spawning migration [45–47] and parental care [48,49], among others [50–52]. These immediate behavioural changes are often linked to state-dependent behaviour, a consequence of physiological disturbance as fish recover from acute stress. The concentration of stress metabolites in fish typically peaks around 1−2 h following the initial stressor [53], and blood lactate levels in rainbow trout subjected to air exposure post-exhaustion have been observed to rise up to 4 h after the stressor [18]. This coincides with the timeframe in which we noted cognitive impairments. This recovery period potentially increases predation risk, as the fish’s diminished executive function and physical coordination could render them more susceptible to predators, and may also negatively impact competition and feeding efficiency [10,54].

Our findings indicate that while air exposure can temporarily impair cognitive abilities in rainbow trout, these effects are reversible and do not persist in the long term. The absence of lasting cognitive damage may be due to the relatively mild hypoxia caused by 60 s of air exposure, consistent with typical C&R fishing scenarios where handling time above water is brief [55]. Although higher temperatures could potentially exacerbate these effects, our study was conducted in cool water (14°C), which may have mitigated the impact of hypoxia [44]. Additionally, the high plasticity of fish brains likely facilitated recovery, preventing long-term damage [28]. Given that rainbow trout are sensitive to low oxygen levels [26], our results are promising for the sustainability of C&R practices.

Our results showed that, in addition to alternations, rainbow trout displayed repetitions (LLLL, RRRR) at similar rates in the FMP Y-maze (figure 1). While alternations are a well-established proxy for working memory across vertebrates, the role of repetitions is less clear and may be linked to stress responses, as seen in other studies [29,32,56]. These repetitions might also reflect species-specific tendencies, such as wall-following behaviour or lateralization [29,33]. Notably, there was no significant effect of treatment on repetition rates (results not shown), suggesting that this behaviour may be a stable pattern in our study fish. As we used farmed rainbow trout, their rearing conditions may influence their frequency of repetitive behaviour. The impact of captivity and rearing environment on cognitive abilities is well-documented, with studies suggesting that the homogeneous nature of captive experiences can reduce behavioural flexibility [57,58]. Therefore, considering these factors is important when interpreting our results, especially as C&R may have a more pronounced effect on cognition in wild fish.

Our study is the first to investigate the effects of C&R stressors on cognitive function in fish. While we observed a significant short-term impact on working memory, the potential for other cognitive effects not evaluated in this study remains. Future research should focus on other species and cognitive abilities, as well as estimating associated fitness effects to enhance our understanding of potential sublethal effects on cognition and improve species-specific management. Moreover, studies considering species-specific responses and increasing water temperatures, which may arise due to climate change [59], will further clarify the cognitive effects of C&R and aid in the development of improved management and conservation strategies.

In conclusion, our results suggest that air exposure results in short-term reversible impairment of cognitive function, with no long-term effects on cognition. C&R should be conducted according to best practices to minimize air exposure [16], as even brief periods of air exposure lead to acute effects. Further research is needed to understand the full scope of potential sublethal effects on cognition and to refine management strategies.

## Data Availability

Data and code are publicly available in the Dryad Digital Repository [60]. Supplementary material is available online [61].

## References

[1] Hilborn R, Branch TA, Ernst B, Magnusson A, Minte-Vera CV, Scheuerell MD, Valero JL. 2003 State of the World’s Fisheries. Annu. Rev. Environ. Resour. **28**, 359–399. (10.1146/annurev.energy.28.050302.105509)

[2] Hutchings JA. 2000 Collapse and recovery of marine fishes. Nature **406**, 882–885. (10.1038/35022565) 10972288

[3] Jackson JB *et al*. 2001 Historical Overfishing and the Recent Collapse of Coastal Ecosystems. Science **293**, 629–637. (10.1126/science.1059199) 11474098

[4] Myers RA, Worm B. 2003 Rapid worldwide depletion of predatory fish communities. Nature **423**, 280–283. (10.1038/nature01610) 12748640

[5] Lewin WC, Arlinghaus R, Mehner T. 2006 Documented and Potential Biological Impacts of Recreational Fishing: Insights for Management and Conservation. Rev. Fish. Sci. **14**, 305–367. (10.1080/10641260600886455)

[6] Lewin WC, Weltersbach MS, Ferter K, Hyder K, Mugerza E, Prellezo R, Radford Z, Zarauz L, Strehlow HV. 2019 Potential Environmental Impacts of Recreational Fishing on Marine Fish Stocks and Ecosystems. Rev. Fish. Sci. Aquac. **27**, 287–330. (10.1080/23308249.2019.1586829)

[7] Post JR. 2013 Resilient recreational fisheries or prone to collapse? A decade of research on the science and management of recreational fisheries. Fish. Manag. Ecol. **20**, 99–110. (10.1111/fme.12008)

[8] Post JR, Sullivan M, Cox S, Lester NP, Walters CJ, Parkinson EA, Paul AJ, Jackson L, Shuter BJ. 2002 Canada’s Recreational Fisheries: The Invisible Collapse? Fisheries **27**, 6–17. (10.1577/1548-8446(2002)0272.0.co;2)

[9] Arlinghaus R, Cooke SJ, Lyman J, Policansky D, Schwab A, Suski C, Sutton SG, Thorstad EB. 2007 Understanding the complexity of catch-and-release in recreational fishing: an integrative synthesis of global knowledge from historical, ethical, social, and biological perspectives. Rev. Fish. Sci. **15**, 75–167. (10.1080/10641260601149432)

[10] Bartholomew A, Bohnsack JA. 2005 A Review of Catch-and-Release Angling Mortality with Implications for No-take Reserves. Rev. Fish Biol. Fish. **15**, 129–154. (10.1007/s11160-005-2175-1)

[11] COOKE SJ, SCHRAMM HL. 2007 Catch‐and‐release science and its application to conservation and management of recreational fisheries. Fish. Manag. Ecol. **14**, 73–79. (10.1111/j.1365-2400.2007.00527.x)

[12] Flink H, Sundblad G, Merilä J, Tibblin P. 2024 Recreational fisheries selectively capture and harvest large predators. Fish Fish. **25**, 793–805. (10.1111/faf.12839)

[13] Holder PE, Wood CM, Lawrence MJ, Clark TD, Suski CD, Weber J, Danylchuk AJ, Cooke SJ. 2022 Are we any closer to understanding why fish can die after severe exercise? Fish Fish. **23**, 1400–1417. (10.1111/faf.12696)

[14] Wood CM, Turner JD, Graham MS. 1983 Why do fish die after severe exercise? J. Fish Biol. **22**, 189–201. (10.1111/j.1095-8649.1983.tb04739.x)

[15] Black EC. 1958 Hyperactivity as a Lethal Factor in Fish. J. Fish. Res. Board Can. **15**, 573–586. (10.1139/f58-030)

[16] Cook KV, Lennox RJ, Hinch SG, Cooke SJ. 2015 FISH Out of WATER: How Much Air is Too Much? Fisheries **40**, 452–461. (10.1080/03632415.2015.1074570)

[17] Cooke SJ, Suski CD. 2005 Do we need species-specific guidelines for catch-and-release recreational angling to effectively conserve diverse fishery resources? Biodivers. Conserv. **14**, 1195–1209. (10.1007/s10531-004-7845-0)

[18] Ferguson RA, Tufts BL. 1992 Physiological Effects of Brief Air Exposure in Exhaustively Exercised Rainbow Trout (Oncorhynchus mykiss): Implications for 'Catch and Release' Fisheries. Can. J. Fish. Aquat. Sci. **49**, 1157–1162. (10.1139/f92-129)

[19] Lefevre S *et al*. 2017 Re-oxygenation after anoxia induces brain cell death and memory loss in the anoxia-tolerant crucian carp. J. Exp. Biol. **220**, 3883–3895. (10.1242/jeb.165118) 29093186

[20] Cooke SJ, Schreer JF, Dunmall KM, Philipp DP. 2002 Strategies for quantifying sublethal effects of marine catch-and-release angling: insights from novel freshwater applications. Am. Fish. Soc. Symp. 121–134. (10.47886/9781888569308.ch16)

[21] Wilson SM, Raby GD, Burnett NJ, Hinch SG, Cooke SJ. 2014 Looking beyond the mortality of bycatch: sublethal effects of incidental capture on marine animals. Biol. Conserv. **171**, 61–72. (10.1016/j.biocon.2014.01.020)

[22] Nilsson GE, Lutz PL. 2004 Anoxia Tolerant Brains. J. Cereb. Blood Flow Metab. **24**, 475–486. (10.1097/00004647-200405000-00001) 15129179

[23] Larson J, Drew KL, Folkow LP, Milton SL, Park TJ. 2014 No oxygen? No problem! Intrinsic brain tolerance to hypoxia in vertebrates. J. Exp. Biol. **217**, 1024–1039. (10.1242/jeb.085381) 24671961 PMC3966918

[24] Caine D, Watson JD. 2000 Neuropsychological and neuropathological sequelae of cerebral anoxia: a critical review. J. Int. Neuropsychol. Soc. **6**, 86–99. (10.1017/s1355617700611116) 10761372

[25] Nilsson GE 1996 Brain and Body Oxygen Requirements of Gnathonemus petersii, a Fish with an Exceptionally Large Brain. J. Exp. Biol. **199**, 603–607. (10.1242/jeb.199.3.603) 9318319

[26] Nilsson GE, Pérez-Pinzón M, Dimberg K, Winberg S. 1993 Brain sensitivity to anoxia in fish as reflected by changes in extracellular K+ activity. Am. J. Physiol. Regul. Integr. Comp. Physiol. **264**, R250–3. (10.1152/ajpregu.1993.264.2.R250) 8447481

[27] Hylland P, Nilsson GE, Johansson D. 1995 Anoxic brain failure in an ectothermic vertebrate: release of amino acids and K+ in rainbow trout thalamus. Am. J. Physiol. Regul. Integr. Comp. Physiol. **269**, R1077–84. (10.1152/ajpregu.1995.269.5.R1077) 7503294

[28] Zupanc GKH. 2009 Towards brain repair: Insights from teleost fish. Semin. Cell Dev. Biol. **20**, 683–690. (10.1016/j.semcdb.2008.12.001) 19111625

[29] Cleal M, Fontana BD, Ranson DC, McBride SD, Swinny JD, Redhead ES, Parker MO. 2021 The Free-movement pattern Y-maze: a cross-species measure of working memory and executive function. Behav. Res. Methods **53**, 536–557. (10.3758/s13428-020-01452-x) 32748238 PMC8062322

[30] Cleal M, Fontana BD, Parker MO. 2021 The cognitive and behavioral effects of D-amphetamine and nicotine sensitization in adult zebrafish. Psychopharmacol. (Berl.) **238**, 2191–2200. (10.1007/s00213-021-05844-5) PMC8292302 33963883

[31] Cleal M, Fontana BD, Hillman C, Parker MO. 2023 Ontogeny of working memory and behavioural flexibility in the free movement pattern (FMP) Y-maze in zebrafish. Behav. Process. **212**, 104943. (10.1016/j.beproc.2023.104943) 37689254

[32] Fontana BD, Alnassar N, Parker MO. 2021 Tricaine Methanesulfonate (MS222) Has Short-Term Effects on Young Adult Zebrafish (Danio rerio) Working Memory and Cognitive Flexibility, but Not on Aging Fish. Front. Behav. Neurosci. **15**, 1–9. (10.3389/fnbeh.2021.686102) PMC8371240 34421552

[33] Fontana BD, Cleal M, Clay JM, Parker MO. 2019 Zebrafish (Danio rerio) behavioral laterality predicts increased short-term avoidance memory but not stress-reactivity responses. Anim. Cogn. **22**, 1051–1061. (10.1007/s10071-019-01296-9) 31342209 PMC6834751

[34] Fontana BD, Cleal M, Parker MO. 2020 Female adult zebrafish (Danio rerio) show higher levels of anxiety‐like behavior than males, but do not differ in learning and memory capacity. Eur. J. Neurosci. **52**, 2604–2613. (10.1111/ejn.14588) 31597204

[35] Cooke SJ *et al*. 2013 The physiological consequences of catch‐and‐release angling: perspectives on experimental design, interpretation, extrapolation and relevance to stakeholders. Fish. Manag. Ecol. **20**, 268–287. (10.1111/j.1365-2400.2012.00867.x)

[36] Harmer AMT, Thomas DB. 2019 pathtrackr: An r package for video tracking and analysing animal movement. Methods Ecol. Evol. **10**, 1196–1202. (10.1111/2041-210x.13200)

[37] Brooks ME, Kristensen K, van Benthem KJ, Magnusson A, Berg CW, Nielsen A, Skaug HJ, Mächler M, Bolker BM. 2017 glmmTMB Balances Speed and Flexibility Among Packages for Zero-inflated Generalized Linear Mixed Modeling. R. J. **9**, 378. (10.32614/rj-2017-066)

[38] Hartig F. 2022 DHARMa: Residual Diagnostics for Hierarchical (Multi-Level/Mixed) Regression Models. CRAN: Contributed Packages. The R Foundation. (10.32614/cran.package.dharma)

[39] Fox J. 2003 Effect Displays in R for Generalised Linear Models. J. Stat. Softw. **8**. (10.18637/jss.v008.i15)

[40] Wickham H. 2016 Ggplot2: elegant graphics for data analysis. Cham, Switzerland: Springer. (10.1007/978-3-319-24277-4_9)

[41] Searle SR, Speed FM, Milliken GA. 1980 Population Marginal Means in the Linear Model: An Alternative to Least Squares Means. Am. Stat. **34**, 216–221. (10.1080/00031305.1980.10483031)

[42] White AJ, Schreer JF, Cooke SJ. 2008 Behavioral and physiological responses of the congeneric largemouth (Micropterus salmoides) and smallmouth bass (M. dolomieu) to various exercise and air exposure durations. Fish. Res. **89**, 9–16. (10.1016/j.fishres.2007.08.008)

[43] Thompson LA, Cooke SJ, Donaldson MR, Hanson KC, Gingerich A, Klefoth T, Arlinghaus R. 2008 Physiology, Behavior, and Survival of Angled and Air‐Exposed Largemouth Bass. North Am. J. Fish. Manag. **28**, 1059–1068. (10.1577/m07-079.1)

[44] Gingerich AJ, Cooke SJ, Hanson KC, Donaldson MR, Hasler CT, Suski CD, Arlinghaus R. 2007 Evaluation of the interactive effects of air exposure duration and water temperature on the condition and survival of angled and released fish. Fish. Res. **86**, 169–178. (10.1016/j.fishres.2007.06.002)

[45] Richard A, Bernatchez L, Valiquette E, Dionne M. 2014 Telemetry reveals how catch and release affects prespawning migration in Atlantic salmon (Salmo salar). Can. J. Fish. Aquat. Sci. **71**, 1730–1739. (10.1139/cjfas-2014-0072)

[46] Havn TB, Uglem I, Solem Ø, Cooke SJ, Whoriskey FG, Thorstad EB. 2015 The effect of catch‐and‐release angling at high water temperatures on behaviour and survival of Atlantic salmon Salmo salar during spawning migration. J. Fish Biol. **87**, 342–359. (10.1111/jfb.12722) 26179562

[47] Lennox RJ, Uglem I, Cooke SJ, Næsje TF, Whoriskey FG, Havn TB, Ulvan EM, Solem Ø, Thorstad EB. 2015 Does Catch‐and‐Release Angling Alter the Behavior and Fate of Adult Atlantic Salmon During Upriver Migration? Trans. Am. Fish. Soc. **144**, 400–409. (10.1080/00028487.2014.1001041)

[48] Suski CD, Svec JH, Ludden JB, Phelan FJS, Philipp DP. 2003 The Effect of Catch-and-Release Angling on the Parental Care Behavior of Male Smallmouth Bass. Trans. Am. Fish. Soc. **132**, 210–218. (10.1577/1548-8659(2003)1322.0.co;2)

[49] HANSON KC, COOKE SJ, SUSKI CD, PHILIPP DP. 2007 Effects of different angling practices on post‐release behaviour of nest‐guarding male black bass, Micropterus spp. Fish. Manag. Ecol. **14**, 141–148. (10.1111/j.1365-2400.2007.00534.x)

[50] Klefoth T, Kobler A, Arlinghaus R. 2008 The impact of catch-and-release angling on short-term behaviour and habitat choice of northern pike (Esox lucius L.). Hydrobiologia **601**, 99–110. (10.1007/s10750-007-9257-0)

[51] Stålhammar M, Linderfalk R, Brönmark C, Arlinghaus R, Nilsson PA. 2012 The impact of catch-and-release on the foraging behaviour of pike (Esox lucius) when released alone or into groups. Fish. Res. **125–126**, 51–56. (10.1016/j.fishres.2012.01.017)

[52] Baktoft H, Aarestrup K, Berg S, Boel M, Jacobsen L, Koed A, Pedersen MW, Svendsen JC, Skov C. 2013 Effects of angling and manual handling on pike behaviour investigated by high‐resolution positional telemetry. Fish. Manag. Ecol. **20**, 518–525. (10.1111/fme.12040)

[53] Cooke SJ, Schreer JF, Wahl DH, Philipp DP. 2002 Physiological impacts of catch-and-release angling practices on largemouth bass and smallmouth bass. Am. Fish. Soc. Symp. **31**, 489–512.

[54] DANYLCHUK AJ, DANYLCHUK SE, COOKE SJ, GOLDBERG TL, KOPPELMAN JB, PHILIPP DP. 2007 Post‐release mortality of bonefish, Albula vulpes, exposed to different handling practices during catch‐and‐release angling in Eleuthera, The Bahamas. Fish. Manag. Ecol. **14**, 149–154. (10.1111/j.1365-2400.2007.00535.x)

[55] Lamansky JA, Meyer KA. 2016 Air Exposure Time of Trout Released by Anglers during Catch and Release. N. Am. J. Fish. Manag. **36**, 1018–1023. (10.1080/02755947.2016.1184200)

[56] Gross AN, Engel AKJ, Richter SH, Garner JP, Würbel H. 2011 Cage-induced stereotypies in female ICR CD-1 mice do not correlate with recurrent perseveration. Behav. Brain Res. **216**, 613–620. (10.1016/j.bbr.2010.09.003) 20837068

[57] Salvanes AGV, Moberg O, Ebbesson LOE, Nilsen TO, Jensen KH, Braithwaite VA. 2013 Environmental enrichment promotes neural plasticity and cognitive ability in fish. Proc. R. Soc. B **280**, 20131331. (10.1098/rspb.2013.1331) PMC3735255 23902903

[58] Arechavala-Lopez P, Caballero-Froilán JC, Jiménez-García M, Capó X, Tejada S, Saraiva JL, Sureda A, Moranta D. 2020 Enriched environments enhance cognition, exploratory behaviour and brain physiological functions of Sparus aurata. Sci. Rep. **10**, 10. (10.1038/s41598-020-68306-6) 32647185 PMC7347547

[59] IPCC. 2023 Climate change 2021: the physical science basis. Contribution of Working Group I to the Sixth Assessment Report of the Intergovernmental Panel on Climate Change. Cambridge, UK: Cambridge University Press. (10.1017/9781009157896)

[60] Flink H, Berge A, Leggieri F, Kolm N, Tibblin P. 2024 Transient cognitive impacts of oxygen deprivation caused by catch-and-release angling [Dataset]. Dryad Digital Repository. (10.5061/dryad.brv15dvk8) PMC11732411 39809327

[61] Flink H, Berge A, Leggieri F, Kolm N, Tibblin P. 2024 Supplementary material from: Transient cognitive impacts of oxygen deprivation caused by catch-and-release angling. Figshare. (10.6084/m9.figshare.c.7585017) PMC11732411 39809327

